# DNA methylation and cognitive aging

**DOI:** 10.18632/oncotarget.4215

**Published:** 2015-05-20

**Authors:** Xiangru Xu

**Affiliations:** ^1^ Max Planck Institute for Biology of Ageing, Cologne, Germany; ^2^ Department of Anesthesiology, Yale University School of Medicine, New Haven, CT, USA

**Keywords:** epigenetics, neurodegeneration, bisulfite DNA methyl-sequencing, transcriptional regulation, neuronal genes

## Abstract

With ever-increasing elder population, the high incidence of age-related diseases such as neurodegenerative disorders has turned out to be a huge public concern. Especially the elders and their families dreadfully suffer from the learning, behavioral and cognitive impairments. The lack of effective therapies for such a horrible symptom makes a great demanding for biological mechanism study for cognitive aging. Epigenetics is an emerging field that broadens the dimensions of mammalian genome blueprint. It is, unlike genetics, not only inheritable but also reversible. Recent studies suggest that DNA methylation, one of major epigenetic mechanisms, plays a pivotal role in the pathogenesis of age-related neurodegenerations and cognitive defects. In this review, the evolving knowledge of age-related cognitive functions and the potential DNA methylation mechanism of cognitive aging are discussed. That indicates the impairment of DNA methylation may be a crucial but reversible mechanism of behavioral and cognitive related neurodegeneration. The methods to examine the dynamics of DNA methylation patterns at tissue and single cell level and at the representative scale as well as the whole genome single base resolution are also briefly discussed. Importantly, the challenges of DNA methylation mechanism of cognitive aging research are brought up, and the possible solutions to tackle these difficulties are put forward.

## HIPPOCAMPUS IS A KEY REGION IN BRAIN COUNTING FOR AGE-RELATED FUNCTIONAL DECLINE IN LEARNING, MEMORY AND COGNITION

The hippocampus is an extremely important component in the brain and is closely associated with the cerebral cortex for learning, memory and cognitive functions. Two major functions of the hippocampus are the storage and interpreter of spatial information, and a mediator of consolidation of short-term memory into long-term memory [1]. Nearly any structural, metabolic or psychological disturbances affecting these areas may result in cognitive behavioral abnormalities such as dementia [2]. Especially, during the normal aging process, humans and animals experience age-related memory and cognitive impairments [1, 3-4]. It was initially thought that the major impact to the etiology of hippocampus function decline with age was a massive loss of neurons and substantial changes in neuronal morphology in pyramidal cell layers [5-7]. However, when it was possible to eliminate many confounding factors of the previous studies, this was proved to be a misconception [8]. As a matter of fact, neuron numbers and morphologies do not change considerably with normal aging in the hippocampus, suggesting that the functional weakening of hippocampal neurons with age is the crucial alteration, which may result from defects in synapse functions, or, in other words, the neuronal plasticity. The mechanisms involved in the regulation of neuronal plasticity in aging as well as other neurological disorders are thus believed to support cognitive functions [8, 9]. Maintenance of long term potentiation (LTP), a cellular indicator of brain cognitive function, requires gene expression and *de novo* protein synthesis [10]; therefore, changes of gene expression/function in neurons are expected to take place with functional deterioration of learning, memory and cognition. Changes in gene sequence as a cause of gene dysfunction leading to mental health disorders were attracted most attention in last few decades, however, sequence changes/variations explain only a small portion of the clinical cases such as Alzheimer's and Parkinson's diseases [11-13]. We and others have found altered synaptic plasticity gene expression in the hippocampus and frontal cortex neuronal cells/tissues with advancing age and age-related neurodegenerative diseases, though molecular mechanisms underlying this altered gene expression are largely unknown [14-16].

## HETEROGENEITY OF HIPPOCAMPAL SUBREGIONAL NEURONS AND THEIR RESPONSES TO AGING

**Figure 1:**
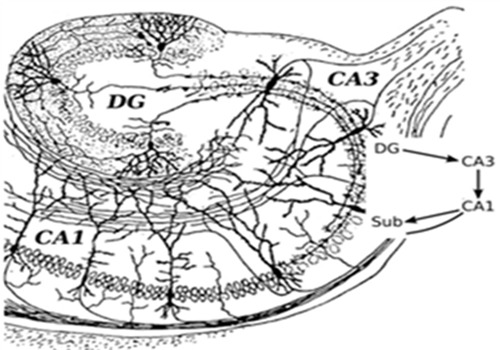
Basic circuit of the hippocampus subregions DG: dentate gyrus. CA1: cornu ammonis 1. CA3: cornu ammonis 3.

It is important to understand that the hippocampus is not a unitary structure—there are three primary cell groups within the hippocampus that combine to make up an internal circuit (Figure 1, adapted from Santiago Ramón Cajal's drawing [17]). These include subregions of cornu ammonis 1 (CA1) and cornu ammonis 3 (CA3) with pyramidal cells and dentate gyrus (DG) with granule cells. Information mostly travels uni-directionally through the hippocampus, beginning with inputs from the entorhinal cortex to the dentate gyrus, then from the dentate gyrus to the CA3 layer, then to the CA1 layer and back to the entorhinal cortex. Other outputs go to several areas of the brain including septal areas and the hypothalamus. High-throughput gene expression investigations in hippocampal CA1, CA3 and DG show regional disparity in response to age and reduced food intake relates to differences in vulnerability to stressors, the availability of neurotrophic, and cell survival mechanisms, and differences in cell function[18, 19]. Major types of hippocampus subregional neurons including CA1 pyramidal neurons, CA3 pyramidal neurons, and DG granule neurons have been studied extensively, and are believed to play central roles for learning and memory and cognitive functions of the hippocampus. Since hippocampal neurons are the main effectors of age-associated neurodegeneration. More importantly, CA1 and CA3 pyramidal neurons are more susceptible to neurodegenerative disorders such as Alzheimer's disease, whereas granule neurons in DG are more vulnerable to age-related damage [1, 20-21]. The mechanism of this selective subregion neuronal vulnerability in hippocampal aging and age-related disorders is unknown yet.

Neuronal recordings from the hippocampus of adult rats reveal that when a rat explores an environment, pyramidal and granule cells show patterned neural activity that is highly correlated with a rat's position in space [22, 23]. Between 30% and 50% of CA1 pyramidal cells show place-specific firing in a given environment [24]. The first demonstration that hippocampal pyramidal cells are functionally heterogeneous in relation to the generation of theta-band oscillation and synchrony was reported with intracellular recordings, which were made in the dorsal hippocampal formation of urethane-anesthetized rats. CA1 pyramidal cells formed theta-related subsets of phasic theta-ON cells and tonic theta-ON cells and non-theta-related subsets of simple spike discharging cells, complex spike discharging cells and “silent” cells. Similar findings were evident for CA3 pyramidal cells [25]. In response to aging, as the number and morphology of cells for the most part do not change, impairment of neuronal functions will further extend the complexity and heterogeneity of neurons in the hippocampus. There is currently little information available on the molecular basis of functional and structural variability between and within different populations of hippocampal neurons. In addition, the variation of gene expression in these cells are believed to be increased with age from cell to cell [26, 27], and the further investigation is urgently needed. Again, the regulation mechanisms underlying this altered gene expression at subregional neuron population and single cell levels are not known.

## THE PLASTICITY OF COGNITIVE AGING

Favorable interventions such as dietary restriction, physical exercise, and enriched environment have been identified as potential means to slow brain ageing and forestall neurodegeneration and cognition, and act in part by increasing BDNF expression and enhancing neurogenesis in hippocampus [28-32]. Inhibiting target of rapamycin (TOR) activity acts to extend lifespan in a range of model organisms, including yeast, worms, and flies [33-37]. Evidence also indicates that mechanistic TOR (mTOR) plays a key role in regulating mammalian lifespan. Treatment of mice with rapamycin at 9 months and 20 months of age all results in an extension of lifespan [38-39]. Rapamycin treatment of mice also achieves an improvement of age-related brain cognition, and abolishes cognitive deficits and reduces the level of amyloid-beta, a widely blamed culprit for neuronal death that causes neuronal degeneration in the hippocampus, in a mouse model of Alzheimer's disease [40-43]. Using a genetic model (mTOR^Δ/Δ^) of reduced mTOR expression, Finkel lab found that reducing mTOR activity at 25% of wild-type levels produces a significant increase (20%) in overall lifespan and a tissue-specific age-related functional preservation in many but not all tissues. The brain was one area where reducing mTOR activity appeared to have a marked functional benefit including spatial learning activity [44].

## DNA METHYLATION AND COGNITIVE AGING

**Figure 2:**
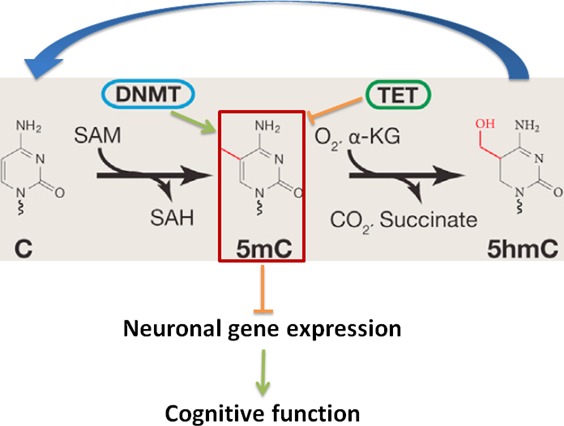
DNA Cytosine(C) modification pathway that includes cytosine methylation (5mC) by DNMTs and the demethylation of 5mC by TETs regulates neuronal gene expression, and thereby cognitive functions SAM: S-adenosylmethionine; SAH, S-adenosylhomocysteine.

DNA cytosine methylation, involving the addition/removal of a methyl group to/from the 5 position of the cytosine pyrimidine ring, is one of main epigenetic mechanisms in higher eukaryotes including plants, rodents, human, and plays a key role in maintaining genome stability and regulating gene expression [45-48]. It has been long believed that DNA methylation, a gene transcription regulation mechanism, does not exist in yeast, worm and fly because the DNA methyltransferases (DNMTs) had been lost during evolution millions of years ago for those lower organisms [49-51]. There are four major dynamic waves of DNA methylation that occur throughout the life of the organism: (i) erasure in primordial germ cells, (ii) parental-specific establishment in the germ line, (iii) selective maintenance during pre-implantation and reprogramming, and (iv) general life-long maintenance. To establish and maintain DNA methylation, nature has evolved an enzymatic toolbox for altering cytosine within the genome. Methylation of cytosine relies on several catalytically active Dnmts. Dnmt1 is a maintenance methyltransferase that copies the pre-existing methyl marks during DNA replication. In contrast, Dnmt3a and Dnmt3b catalyze *de novo* DNA methylation during development and other pathophysiological conditions. The reverse of this process, DNA demethylation, is much less studied. But several studies imply that Tet enzymes are involved in both global- and locus-specific DNA demethylation, by catalyzing the conversion of 5-methylcytosine to 5-formylcytosine and 5-carboxylcytosine. Those modified cytosines are then completely removed by thymine-DNA glycosylase-initiated DNA base excision repair [52-54]. The dynamics of genome-wide DNA methylation are regulated by DNA methyltransferases (Dnmts) including Dnmt1, Dnmt3a and Dnmt3b. The key DNA demethylation enzymes are ten-eleven translocation (Tet) family enzymes such as Tet1, Tet2 and Tet3 (Figure 2). The decline of overall DNA methylation has been associated with cell and tissue aging including brain [55-57]. The loss of Dnmt1 and Dnmt3a in the adult brain leads to cognitive deficits in mice [48, 58-59]; and mutant Tet1 animal exhibits abnormal hippocampal long-term depression and impaired memory extinction [60]. In humans, mutations in DNMT1 are associated with a form of neurodegenerative disease [61]. These studies suggest that impairment of DNA methylation may play a crucial role and be a fundamental mechanism in regulating mouse learning memory and cognition.

Unlike genetics, epigenetics is not only inheritable but also reversible. Therefore, strategies aimed to reverse age-associated epigenetic alterations may lead to the development of a novel therapeutic intervention to delay aging or alleviate symptoms of devastating age-associated diseases. Recently, a report states that transient over-expression of Dnmt3a2, a Dnmt3a isoform, in mouse hippocampus can restore age-associated cognitive deficits and inhibition of hippocampal Dnmt3a2 expression by shRNAi leads to a damage in young mouse cognitive behavioral [62-63]. It suggests that DNA methylation plays a crucial role for maintaining normal hippocampal function and confers an epigenetic mechanism for learning memory and cognition. Though the further works including whole genome DNA methyl-sequencing and genetic manipulation of Dnmt3a2 in mouse brain are needed immediately to elucidate how DNA methylation affects the expression of synaptic plasticity genes and thereby impacts the learning memory and cognition.

## OTHER EPIGENETIC FACTORS AND COGNITIVE AGING

Conrad Waddington in 1940s proposed a term “epigenetics” to describe interactions of genes with their environment during development [64]. It has emerged as a possible mechanism controlling the gene expression and a potential causative factor of brain aging and other memory and cognitive abnormalities until recently [65]. Epigenetic regulation is primarily mediated by DNA methylation and posttranslational modifications of nucleosomal histones. Post-translational modifications of nuclear histone tails represent one of basic molecular epigenetic mechanisms that alter chromatin structure and accessibility of DNA, and influence the gene expression and potentially cellular/organismal phenotypes [66-68]. It has been established that regulation of chromatin structure through post-translational modification of histone proteins is important for the induction of synaptic plasticity and formation of long-term memory [65]. For example, chromatin remodeling via histone acetylation plays a crucial role in regulating synaptic and cognitive function in aging and age-related neurodegeneration. Increasing histone acetylation by inhibition of histone deacetylase (HDAC) enhances gene transcription and improves hippocampal LTP, a cellular mechanism that underlies learning and memory [69-70]. The trimethylation of histone H3 at lysine 4 (H3K4), an active mark for transcription, is upregulated in hippocampus following contextual fear conditioning [71]. Not limited to histone modifications, non-coding RNAs and their networks also underlie cognitive disorders across the lifespan and favorable interventions [72-73].

## PROFILING THE GENOME-WIDE DNA METHYLATION AT TISSUE, CELL POPULATION AND INDIVIDUAL CELL

Accumulated evidence suggested that DNA methylation regulation is critical for maintaining normal hippocampal function and confers an epigenetic mechanism for age-related learning memory and cognition [62-64]. It is thus urgently needed appropriate methods to measure the dynamics of genome-wide DNA methylation in neuronal tissues/cells. Weber M. et al. in 2005 was first described a method, methylated DNA immunoprecipitation (MeDIP)-chip, to assess the genome-wide DNA methylation [74]. It consists of enriching methylated DNA fragments through an antibody against 5-methylcytosine (5mC), and detecting the purified fraction of methylated DNA with high-throughput DNA methylation arrays. MeDIP-chip (e.g., mouse and human promoter CpG arrays) can be used to map the dynamic alterations of genome-wide promoter CpG methylation in aging tissues [45]. Almost in the same time, Meissner et al first reported a reduced representation bisulfite sequencing (RRBS) method to dissect the methylome of mammalian cells [75]. RRBS is based on the lack of even distribution and the fact that CpG sites within the mammalian genome tend to cluster together as CpG islands (CGIs) that are usually located close to the promoters of known genes [76]. So, firstly cutting the genome into small fragments by a restriction enzyme that recognizes CpG and its flanking sequences, then most of the CGIs will be collected and sequenced with high coverage even with a lower numbers of total sequencing reads (e.g., ~50 million reads). RRBS has led to important findings regarding global methylation and demethylation process during early developmental stages [77]. Lister et al. described a whole-genome bisulfite sequencing (WGBS) to map DNA methylations at single base resolution [78]. It is currently the gold standard for DNA methylome measurement and it provides coverage for more than 90% of the approximately 28.7 million CpGs in the human genome [79]. However, it demands a much higher sequencing reads, the minimum request for sequencing reads coverage is about 30X genome size. Those methods not only are good for tissue level study but also can be used for cell population interrogation for DNA methylome.

As abovementioned example that neurons from one type of populations are possibly different from one to another, one is more stable and resistant to stressors but another could be vulnerable to the same stressors. Single cell transcriptome analysis has been achievable and is proved as a powerful tool to understand the variation among same type of cells [80]. The methods to examine the genome-wide DNA methylation at single level were also essentially desired. Guo et al. reported a methylome analysis method that enables single-cell at single-base resolution DNA methylation analysis based on reduced representation bisulfite sequencing (scRRBS) [81]. scRRBS is integrated all of the experimental processes in a single-tube reaction without including any purification steps prior to the bisulfite conversion step, since the multiple purification steps are the major problem for massive loss of DNA. This technique is sensitive and can detect the methylation status of up to 1.5 million CpG sited within the genome of an individual embryonic stem cell [82]. While Smallwood et al. described a single-cell bisulfite sequencing (scBS-seq) method which can be applied to accurately measure DNA methylation at up to 48.4% of CpG sites [83]. In BS-seq protocols, bisulfite treatment is performed first then sequencing adaptors that are ligated to fragmented DNA minimizing the DNA loss from single cell. In brief, those are all powerful tools for us to map out the DNA methylation at tissue, cell population and single cell level facilitating a better understanding the mechanism of DNA methylation in neuronal gene regulation and thereby the cognitive function.

## PERSPECTIVES AND CHALLENGES TO PIECE OUT THE ROLE OF DNA METHYLATION IN TRANSCRIPTIONAL REGULATION AND ITS IMPACT ON AGE-RELATED COGNITIVE FUNCTION

### The complexity of transcriptional regulation by DNA methylation

Learning and memory are two intimately linked cognitive processes that stem from interactions between genes and the environment (experience). These cognitive functions have also been associated with changes in gene expression, and a number of genes have been found to enhance or impair learning and memory. Dysregulation of synaptic plasticity genes, such as brain-derived neurotrophic factor (Bdnf), cAMP response element binding (Creb) and activity regulated cytoskeletal-associated protein (Arc) have been strongly correlated with mammalian brain aging and cognitive decline. For instance, polymorphisms in the human BDNF gene have been associated with memory and hippocampal function [84]. Bdnf-deficient mice display premature age-associated decrements [85]. Hippocampus-specific deletion of Bdnf in adult mice impairs spatial memory and extinction of aversive memories [86]. Mice with Creb deficiency have a mild cognitive impairment, and exhibit a deficit in condition-dependent learning and memory tests [87].

**Figure 3:**
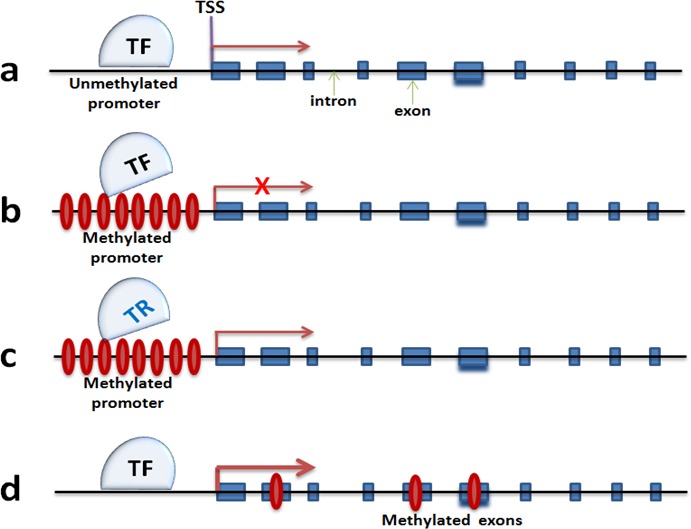
DNA methylation mechanisms in regulating neuronal gene expression **a.** and **b.** are dominant theories that promoter methylation silences gene transcription by blocking the landing of transcription complex. **c.** proposes an activation of gene transcription by a failure landing of neuronal gene repressors. **d.** is a newly discovered mechanism that exon methylation correlates with gene expression augmentation. TF: transcription factor; TR: transcription repressor; TSS: transcription start site.

Expression of Arc, a neuronal activity-relevant gene, decreases with age, and this decreased expression correlates with DNA hypermethylation of its promoter ([88], Figure 3a and 3b). it is also known that upregulation of Dnmt3a2 in hippocampus can restore age-related cognitive function, though it is not known yet how precisely Dnmt3a2 contributes to cognitive function [62]. Higher levels of Dnmt3a2 presumably result in an increase of DNA methylation of Dnmt3a2 target genes. The expression of synaptic plasticity genes like Bdnf, c-Fos and Arc are increased significantly with over-expression of Dnmt3a2. This sounds counterintuitive on the basis of the traditional view that DNA methylation is associated with transcriptional repression (Figure 3a and 3b). However, there are reports suggesting that exonic DNA methylation may serve as a transcriptional activator that triggers gene transcription ([89], Figure 3d). This also could be caused by methylation-associated blocking of transcriptional repressor (TF) such as neuron restrictive silencer factor (Nrst/REST), which has been reported to be involved in transcriptional repression of Bdnf. However, BDNF expression will be released after NRST/REST being inhibited by the promoter methylation ([90], Figure 3c). This is just one example, not even mentioning the impact of DNA methylation on other DNA elements such as the enhancer, and the non-coding RNAs including long non-coding RNA and miRNA which may result in regulation of neuronal gene expression [91]. Taken together, it raises additional layers of complexity for understanding the role of DNA methylation in neuronal gene expression regulation. Deep sequencing methods such as BS-DNA-methyl-seq and RNA-seq at neuronal tissue and cell level will be an effective tool to better understand this complexity.

### The functional significance of DNA methylation

Furthermore, the animal models as well as the cutting-edge genetic manipulation approaches should be employed to determine the biological significance of DNA methylation on learning, memory and cognitive function [92]. For example, a constitutive and an inducible forebrain neuron-specific Dnmt3a2 transgenic mouse line could be generated to test if higher level Dnmt3a2 in neurons can enhance/restore mouse learning, memory and cognitive functions. In contrary, a Dnmt3a2 conditional forebrain neuron-specific knock-out mouse line will also be useful to measure if Dnmt3a2 is essential for maintaining learning and cognition. Besides, genome-wide analysis of DNA methylation landscape and transcriptome in parallel in neurons at various conditions including age and expression level of Dnmt3a2 via bisulfite sequencing and RNA-seq would be great to identify neuronal targets of Dnmt3a2.

### The coordination of DNA methylation and histone modification

The last but not least is to understand the coordination of DNA methylation and histone modification in regulation of neuronal gene expression and learning, memory and cognition. In addition to the significance of DNA methylation to cognitive function, several laboratories including us discovered that the acetylation and methylation of histones (e.g., H4K12ac, H3K9me3 and H3K27me3) are playing critical roles in age-related behavioral and cognitive functions [70-71, 73, 93-94]. The Polycomb target genes provide the first example of how histone modification and DNA methylation work together to achieve silencing, and the mechanism of transcriptional repression in such a case involves the generation of local heterochromatin- the histone methyltransferase EZH2, H3K27me3 and the methylated DNA ‘landing dock’ sites [95]. It's also reported that both histone methylation, such as methylation of H3K9 mediated by by G9A, and DNA methylation is essential for repression of pluripotency-associated genes including *Oct4* and *Nanog* during embryonic cell differentiation [96]. Thus, it will be interesting and necessary to clarify whether DNA methylation and histone modification work synergistically or independently to regulate the learning and cognitive functions in the mouse model.
